# Primary Nasal Tuberculosis Masquerading as Granulomatosis With Polyangiitis: A Case Report of Diagnostic Dilemma

**DOI:** 10.7759/cureus.49649

**Published:** 2023-11-29

**Authors:** Khoo Shu Jiun, Syafiqah Kamel, Kanivannen Arasu, Khairunnisa M Zuhaidi, Avatar Singh Mohan Singh

**Affiliations:** 1 Otorhinolaryngology - Head and Neck Surgery, Taiping Hospital, Taiping, MYS; 2 Pathology, Taiping Hospital, Taiping, MYS

**Keywords:** wegener’s granulomatosis, tuberculosis in sinonasal region, head and neck tuberculosis, granulomatosis with polyangiitis, primary nasal tuberculosis

## Abstract

Primary nasal tuberculosis (TB) is a rare disease even in areas with high TB burden, possibly attributed to the protective mechanism of sinonasal mucosa. Its symptoms are subtle and can be mistaken for other granulomatous inflammatory conditions. We would like to report a case of a 70-year-old Indian lady who underwent a successful left endoscopic dacryocystorhinostomy three years ago and presented with recurrent left epiphora. During nasal endoscopy, multiple ulcerative masses with crusting were detected over the left nasal vestibule, anterior nasal septum, left inferior, and middle turbinate. Biopsy of the nasal mass revealed granulomatous inflammation without caseating necrosis. Initially, all TB-related tests were negative. As the patient had granulomatous nasal lesions with microscopic haematuria, granulomatosis with polyangiitis (GPA) was suspected. Regrettably, the patient did not respond to treatment. A repeated tissue culture at a later stage finally detected mycobacterium tuberculosis without the presence of pulmonary tuberculosis. Considering the current TB prevalence in the Southeast Asian region, it is crucial for otorhinolaryngologists to be aware of primary nasal TB when encountering unusual head and neck lesions, even in the absence of pulmonary TB.

## Introduction

Sinonasal tuberculosis (TB) usually occurs secondary to pulmonary TB by inhalation of infected particles and rarely presents as a primary lesion [1]. The first case of nasal TB was reported by Giovanni Morgani in 1761 while reporting the autopsy findings of a young gentleman who had pulmonary TB with ulcerations of the nose, soft palate, and nasopharynx [2]. The first primary nasal TB was only reported to the Pathological Society of London by Clarke in 1852 [2]. Later in the 18th century, Herzog described a total of 20 primary nasal TB among 80 cases of nasal TB [2]. In a 20th-century medical literature published in 1997, Butt reported only 35 cases of nasal TB in the last 95 years [3]. A 10-year retrospective study conducted in India from 1995 to 2994 revealed that, out of 165 cases of TB in the head and neck region, only one was nasal TB [4]. Diagnosis of nasal TB can be very challenging as it is rare and only shows subtle signs and symptoms that can mimic other non-specific nasal inflammatory conditions. This can lead to a diagnostic dilemma and delay in initiating definitive treatment.

In this case report, we describe a 70-year-old Indian lady who was found to have left-sided nasal lesions, managed over a period of one year in our clinic. We also discussed the clinical case, diagnostic workup, and the importance of histopathological and microbiological diagnosis for her management planning.

## Case presentation

A 70-year-old Indian lady with underlying type II diabetes mellitus, hypertension, and dyslipidaemia underwent a successful left endoscopic dacryocystorhinostomy (EDCR) three years ago and was discharged well. She presented back to us with recurrent left epiphora for three months. She had no symptoms of dacryocystitis, nasal blockage, anosmia, or rhinorrhea. She also had no epistaxis, no prolonged cough, or hemoptysis.

**Figure 1:**
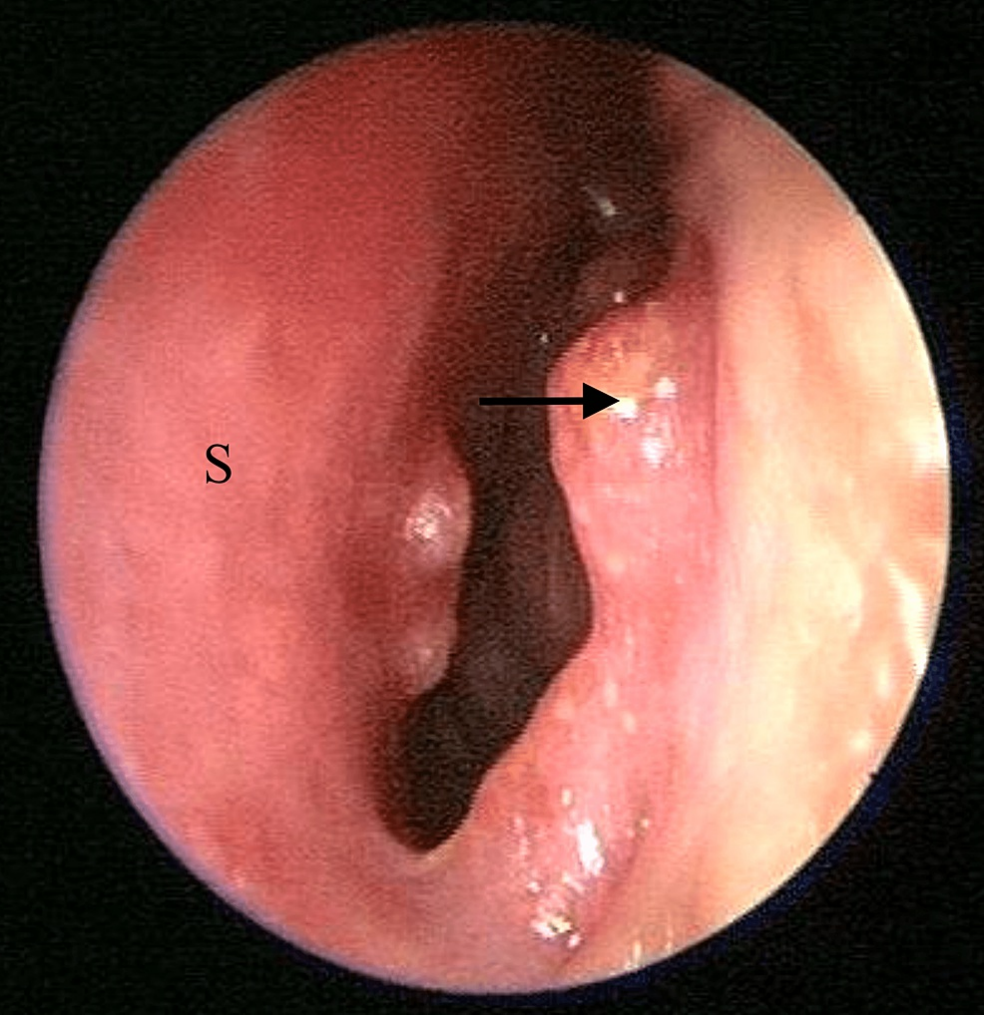
Left nasal vestibule mass (arrow) S: Septum

**Figure 2:**
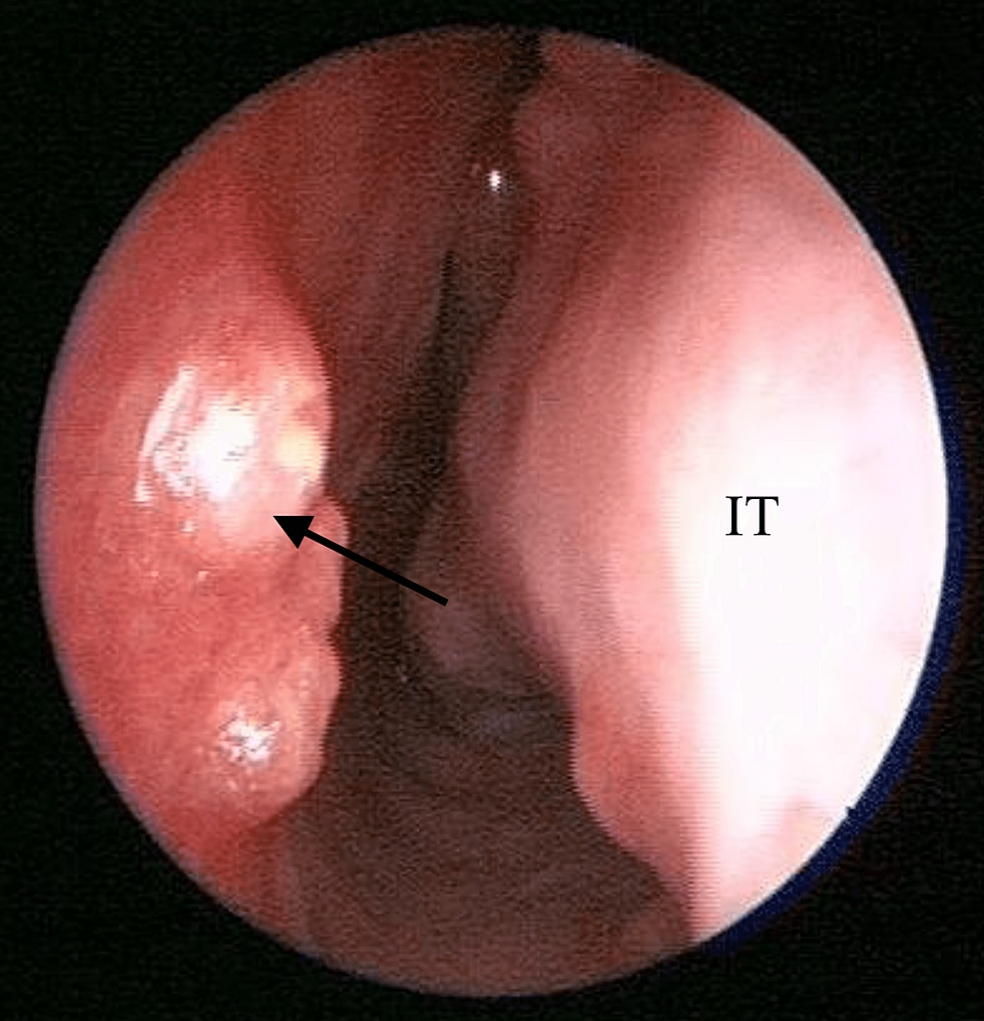
Nasal septum mass lesion IT: Inferior Turbinate

**Figure 3:**
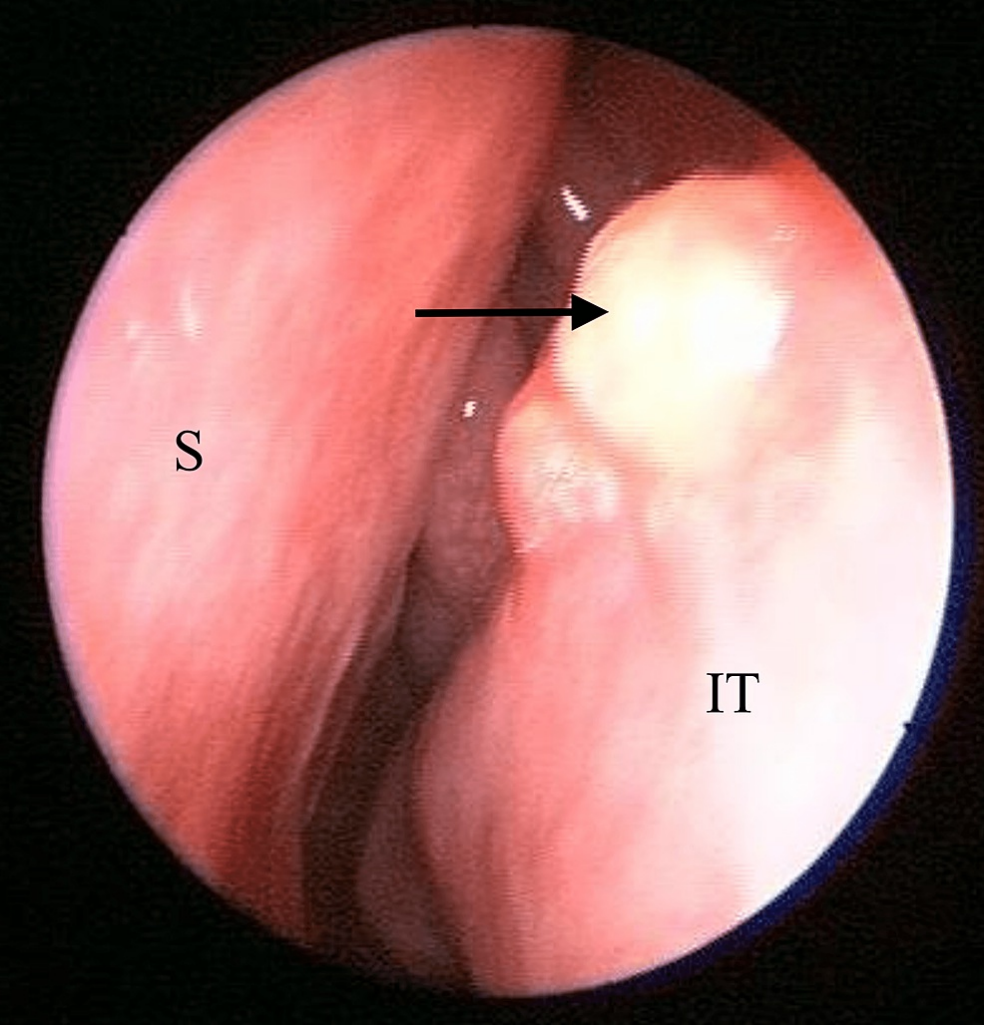
Left inferior turbinate (IT) lesion (arrow)

**Figure 4:**
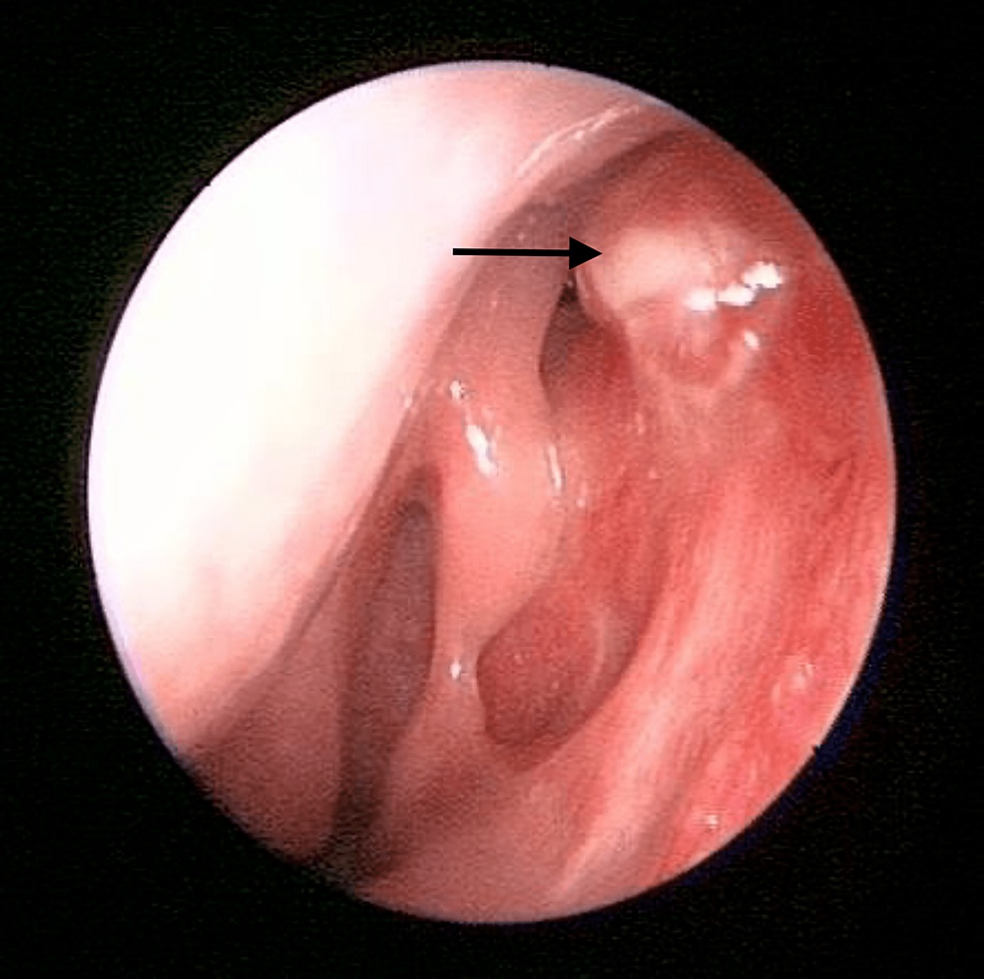
Left middle turbinate lesion (arrow) with evidence of previous EDCR done EDCR: Endoscopic Dacryocystorhinostomy

Upon examination, there was no sign or symptoms of dacryocystitis. Nasal endoscopy of the left nasal cavity showed multiple irregular, sessile masses with crusting over the left nasal vestibule, anterior nasal septum, inferior turbinate, and middle turbinate (Figures 1-4). Other head and neck examinations and systemic examinations, including lung and renal, were unremarkable.

**Figure 5:**
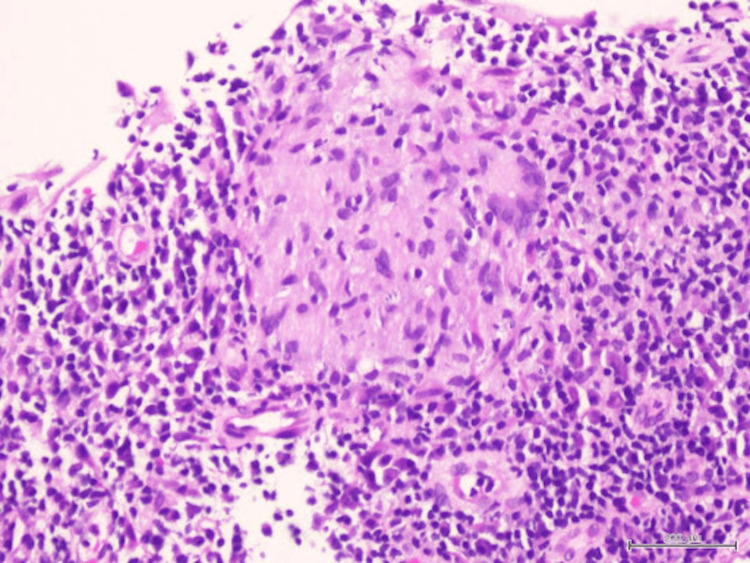
Well-formed epithelioid granuloma without central necrosis

**Figure 6:**
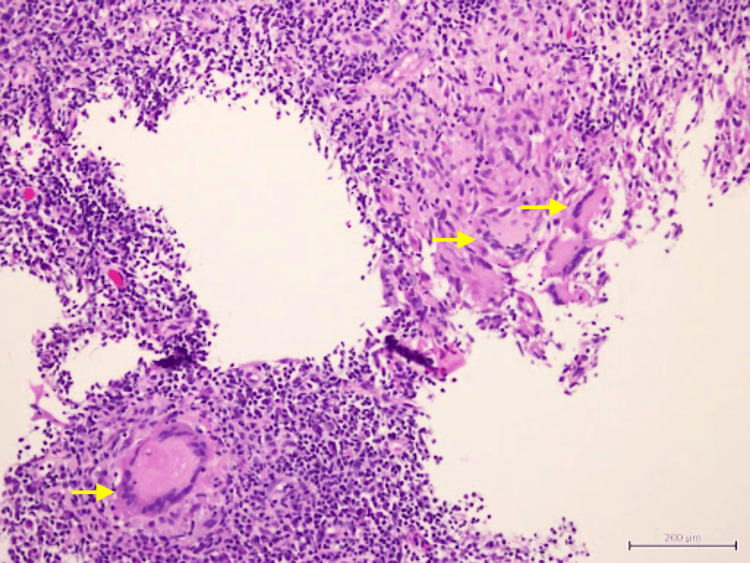
Langhans-type multinucleated giant cells (yellow arrow)

Biopsy of the nasal masses revealed granulomatous inflammation with occasional Langhans multinucleated giant cells. No caseating necrosis was seen (Figures 5, 6). Ziehl Nelson staining was negative, and no fungal elements were identified with periodic acid-Schiff and Grocott’s methenamine silver stain. Further GeneXpert polymerase chain reaction assay of Mycobacterium tuberculosis complex was reported as negative, and acid-fast bacilli (AFB) staining of her sputum was negative as well.

**Figure 7:**
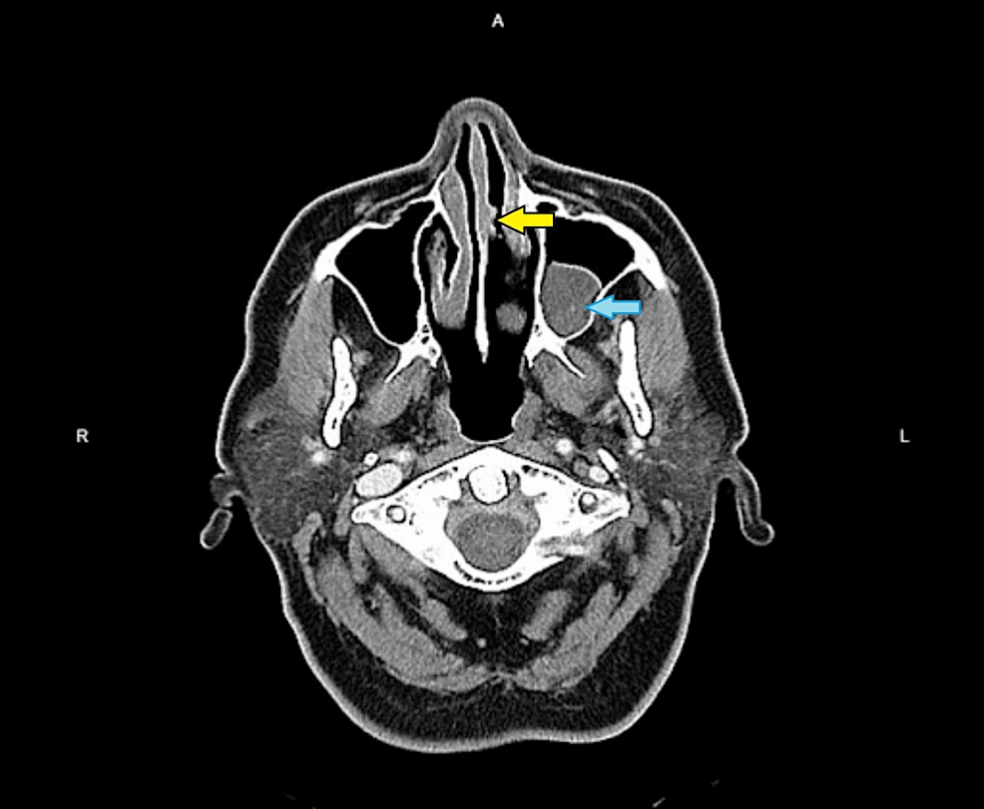
CECT PNS showing a small non-enhancing lesion over the septum (yellow arrow) and left maxillary sinus mucosal thickening (blue arrow)

Blood investigations showed normal full blood count, renal profile, liver function, and normal calcium level. Further viral screening and autoimmune screening, including complement factors 3 and 4, rheumatoid factor (RF), anti-nuclear antibodies (ANA), anti-double stranded DNA (anti-dsDNA), anti-single stranded DNA (anti-ssDNA), anti-neutrophil cytoplasmic antibodies (ANCA), anti-myeloperoxidase (anti-MPO), and anti-proteinase 3 (anti-PR3), were all negative. Her chest X-ray showed a clear lung field. Contrast-enhanced computed tomography of paranasal sinuses (CECT PNS) showed a small non-enhancing lesion over the nasal septum and homogenous mucosal thickening in the left maxillary sinus (Figure 7). MRI brain was done as well, but no significant findings were noted.

GPA was the first differential diagnosis on the list as her urine analysis detected microscopic haematuria. She was then referred to the local rheumatologist and was treated with a limited GPA. Oral prednisolone 30 mg daily for two weeks was administered, and then it was tapered down over a period of 10 weeks. However, her nasal lesion did not respond to the treatment.

**Figure 8:**
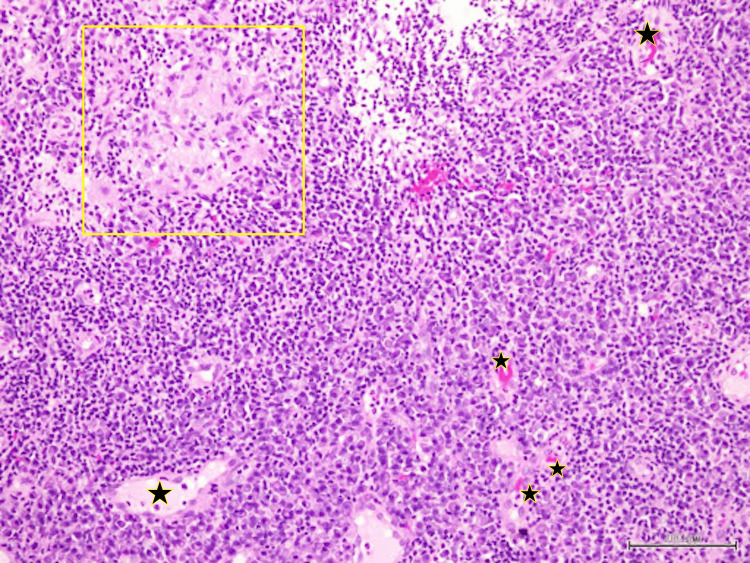
Congested thin-walled blood vessels (black stars) with epithelioid granuloma (yellow box) surrounded by dense amounts of lymphocytes and plasma cells. No features of vasculitis

**Figure 9:**
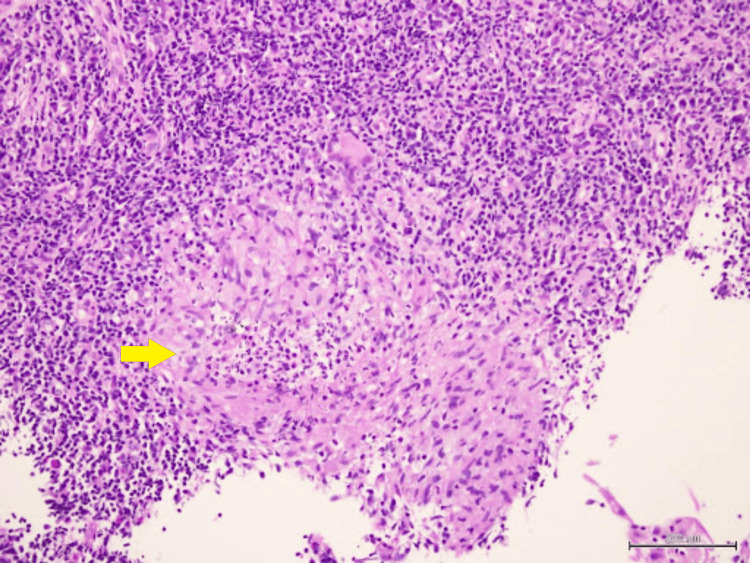
Epithelioid granuloma rimmed by neutrophils, lymphocytes, and plasma cells

**Figure 10:**
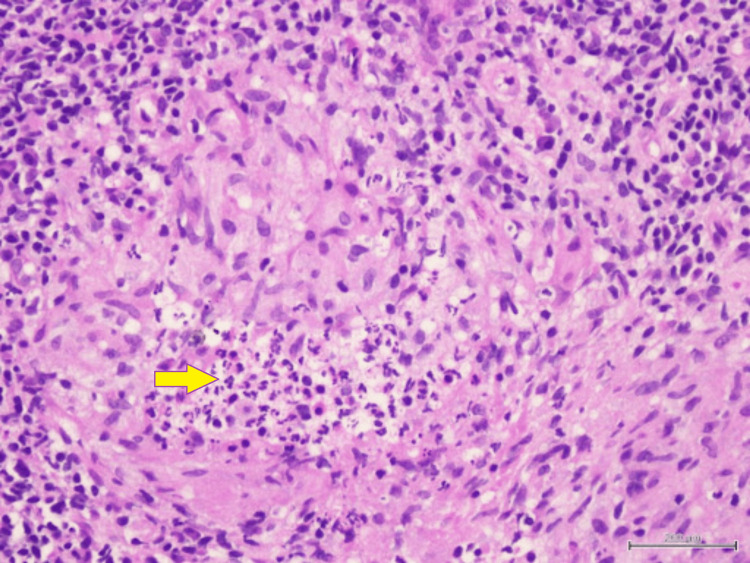
High-power view of epithelioid granuloma with central collection of neutrophils

**Figure 11:**
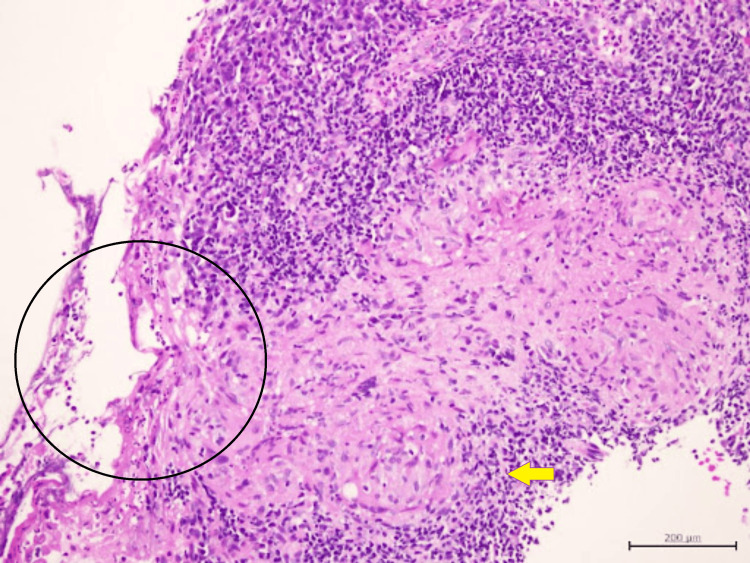
Epithelioid granuloma (yellow arrow) with overlying surface ulceration covered by fibrinopurulent exudate (black circle)

Another tissue biopsy was repeated to look for evidence of vasculitis. The tissue sample was also sent for TB culture and sensitivity, as well as BACTEC. The HPE report revealed granulomatous inflammation with no features of necrotizing (leukocytoclastic) vasculitis, and again no caseous necrosis was seen (Figures 8-11).

**Figure 12:**
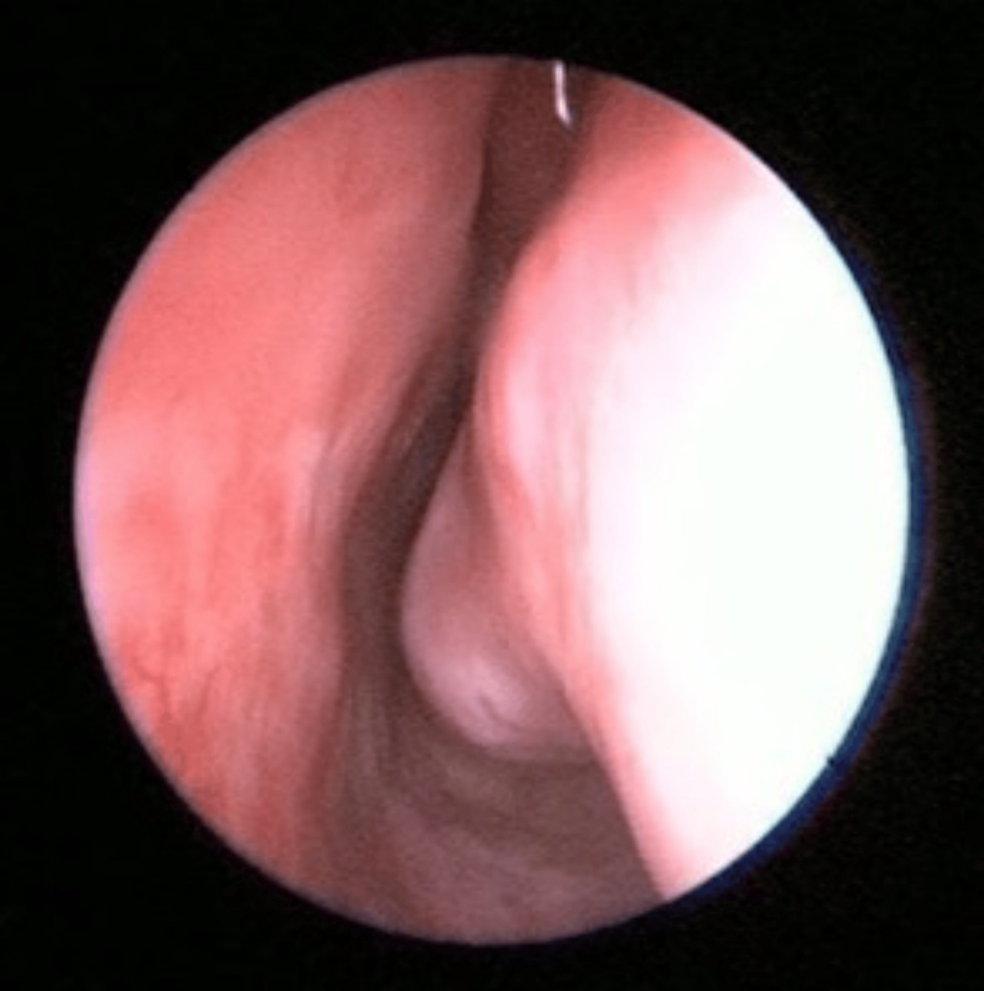
Resolved left nasal septum and inferior turbinate lesions Comparison made with Figures 1, 2

**Figure 13:**
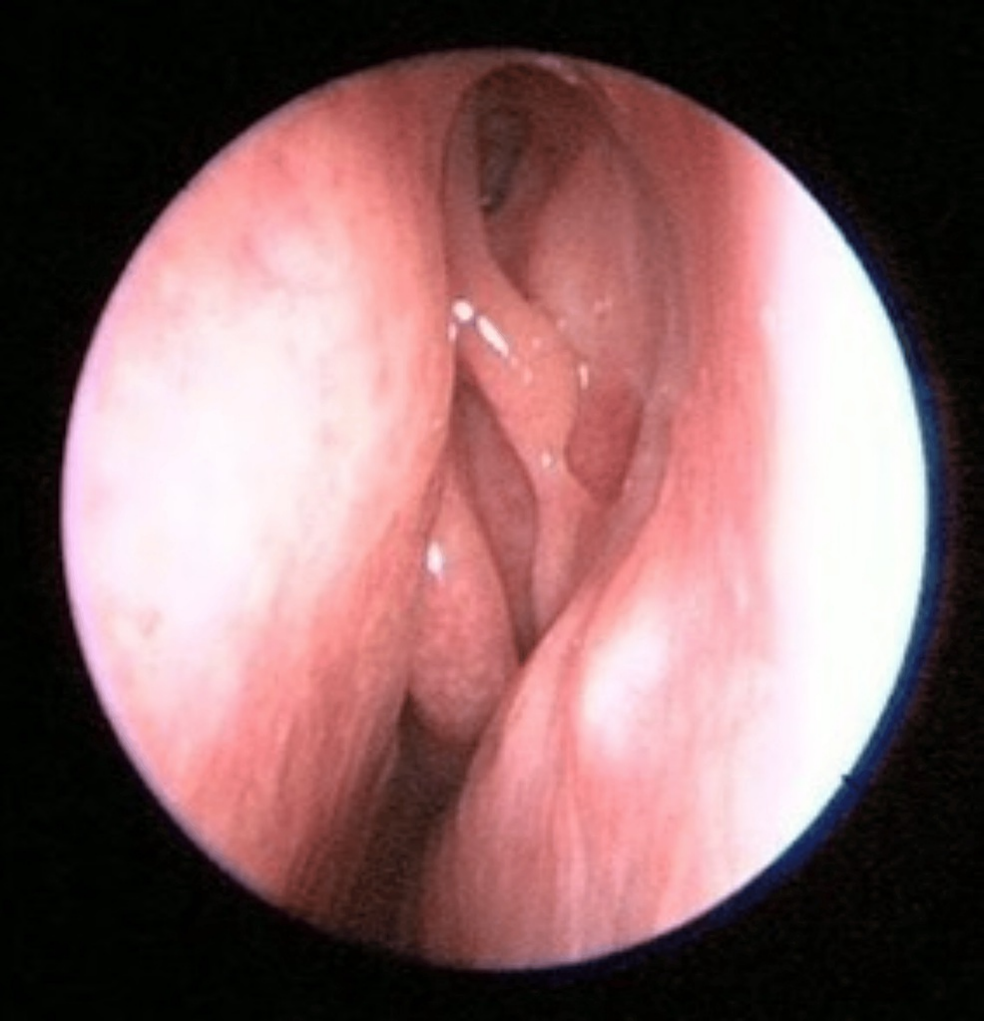
Resolved middle turbinate lesion Comparison with Figure 4

After a long and puzzling diagnosis process, the BACTEC culture test successfully isolated Mycobacterium TB, which proved to be the turning point in resolving the diagnosis dilemma, 12 months after the initial presentation. The case was subsequently managed in cooperation with a respiratory physician. Treatment with isoniazid, rifampicin, pyrazinamide, and ethambutol was instigated. Upon follow-up after completion of anti-TB medication six months later, the patient has shown resolution of the lesions from a nasal endoscope (Figures 12, 13) and complete relief from epiphora.

## Discussion

WHO Global Tuberculosis 2023 has reported a rise in TB incidence in the Southeast Asian (SEA) region, accounting for 48% of the global TB burden with 17% of them being extrapulmonary TB (EPTB) [5]. Although head and neck TB is fairly common in extrapulmonary manifestations, primary nasal TB without pulmonary involvement has always been extremely rare even in countries with high Tb burden such as India [4,6]. Nasal TB is found predominantly among females and in the elderly, especially in people with poor socioeconomic backgrounds [7], which correlates to our patient in the presenting case. The most common routes of infection for nasal TB are usually secondary to pulmonary TB via direct spread, hematogenous, lymphatic route, or in the form of lupus vulgaris in cutaneous lesion [6].

Physiologically, nasal mucosa possesses high resistance to Mycobacterium bacilli due to several defensive mechanisms, including ciliary clearance, bactericidal activity of nasal secretion, mechanical filtering via vibrissae, and intrinsic resistance to mycobacterial infection [8]. In our patient, it is most likely that the nasal mucosa and cilia were compromised due to previous endoscopic dacryocystorhinostomy and immunocompromised state owing to type II diabetes mellitus [9]. However, from initial investigations, the lack of positive findings to support the diagnosis of TB has raised the suspicion of other granulomatous diseases.

The patient was suspected of having granulomatous polyangiitis due to the detection of microscopic hematuria. GPA, previously known as Wegener’s granulomatosis, was a potentially lethal autoimmune vasculitis syndrome involving the respiratory system and renal before the advent of immunosuppressive therapy [10]. Up to 95% of the patients will present first with ORL manifestations, and a quarter of them involve only the sinonasal region [11]. When symptoms of GPA appear only in the head and neck regions, it is diagnosed as "limited GPA." While in more advanced stages, when systemic vasculitis is evident, the term “generalized GPA” is used [12].

Clinical presentations for both nasal TB and GPA are very similar. Patients typically present with nasal obstruction with or without discharge. Epistaxis may occur if the disease is in the active phase. As erosion of the nasal mucosa occurs, crusting develops. When the diseases go into remission, crusting, scarring, and adhesions become more prominent [13]. Ulceration and septal perforation may develop at a later stage, leading to cosmetic changes due to internal destruction. Eye symptoms such as epiphora may occur due to blockage of the nasolacrimal duct or direct orbital invasion [7,14]. The vague differences between presentations of granulomatous diseases have made it crucial to further investigate through tissue biopsy.

From tissue biopsy, the possibility of both local disease and systemic disorder should be considered when granulomatous lesions are found. Granulomatous inflammation is formed due to chronic inflammation that occurs in response to various agents such as persistent infectious pathogens or foreign body reactions [15]. In persistent inflammation, macrophages, derived from activated mononucleocytes, will transform into epithelioid cells and then coalesce into multinucleated giant cells to concentrate immune response (lymphocytes) toward the pathogen, forming what is known as granuloma [16]. Classical TB lesions typically show well-formed spherical granuloma with central caseous necrosis and tend to exhibit Langhans giant cells [16,17]. However, both caseating and non-caseating granulomas have been reported [17]. On the contrary, the hallmark of GPA is necrotizing vasculitis involving medium to small arteries and veins. Inflammatory cells including neutrophils, histiocytes, and lymphocytes infiltrate onto vessel wall, causing focal inflammation and necrosis, sparing the rest [18].

The WHO’s consolidated guidance since 2014 has recommended that Xpert MTB/RIF can be used as a replacement test for testing specific non-respiratory specimens (lymph nodes and tissues) from patients suspected of having EPTB, but with low-quality evidence [19]. It was mentioned that, when compared to the results against culture as a reference standard, the sensitivity of the Xpert system varied widely and ranged from 42% to 100% [19]. To date, TB culture is still regarded as the gold standard for detecting EPTB [19]. Even so, due to the paucibacillary nature of tissue samples, culture negativity in head and neck TB may be as high as 50%-75% [20]. During the robust investigations for a definitive diagnosis for our patient, microbial culture was only sent at a later stage. Fortunately, the tissue biopsy finally detected MTB from the BACTEC radiometric culture.

## Conclusions

A decrease in reported TB cases during the COVID-19 pandemic indicates a rise in undiagnosed and untreated cases, leading to increased community transmission and subsequent rebound of cases in 2022. It is very important that otolaryngologists remain cognisant of primary nasal TB as a potential entity when encountering unusual lesions in the sinonasal region. Although polymerase chain reaction amplification and modern immunological methods have the advantages of more rapid diagnosis and fair performance in diagnosis, the standard TB culture (BACTEC) should also be included in initial investigations in atypical nasal lesions or other tissue biopsy in the head and neck regions. It should be kept in mind that clinical judgment, pathological evidence, repeated biopsies, and meticulous investigations are often necessary to properly diagnose and treat granulomatous nasal lesions.
